# Collagen Hydrolysate Effects on Photodynamic Efficiency of Gallium (III) Phthalocyanine on Pigmented Melanoma Cells

**DOI:** 10.3390/gels9060475

**Published:** 2023-06-09

**Authors:** Vanya Mantareva, Ivan Iliev, Inna Sulikovska, Mahmut Durmuş, Tsanislava Genova

**Affiliations:** 1 Institute of Organic Chemistry with Centre of Phytochemistry, Bulgarian Academy of Sciences, Acad. G. Bonchev, Bl. 9, 1113 Sofia, Bulgaria; 2 Institute of Experimental Morphology, Pathology and Anthropology with Museum, Bulgarian Academy of Sciences, Bl. 25, 1113 Sofia, Bulgaria; taparsky@abv.bg (I.I.); inna_sulikovska@ukr.net (I.S.); 3 Department of Chemistry, Gebze Technical University, 41400 Kocaeli, Turkey; durmus@gtu.edu.tr; 4 Institute of Electronics, Bulgarian Academy of Sciences, Tzarigradsko Chaussee 72, 1784 Sofia, Bulgaria; ts.genova@gmail.com

**Keywords:** bovine collagen hydrolysate and gels, gallium phthalocyanine, pigmented melanoma, normal keratocytes and fibroblasts, mouse embryonal fibroblasts, photodynamic therapy

## Abstract

The conjugation of photosensitizer with collagen seems to be a very promising approach for innovative topical photodynamic therapy (PDT). The study aims to evaluate the effects of bovine collagen hydrolysate (Clg) on the properties of gallium (III) phthalocyanine (GaPc) on pigmented melanoma. The interaction of GaPc with Clg to form a conjugate (GaPc-Clg) showed a reduction of the intensive absorption Q-band (681 nm) with a blue shift of the maximum (678 nm) and a loss of shape of the UV-band (354 nm). The fluorescence of GaPc, with a strong emission peak at 694 nm was blue shifted due to the conjugation which lower intensity owing to reduce quantum yield (0.012 vs. 0.23, GaPc). The photo- and dark cytotoxicity of GaPc, Glg and GaPc-Clg on pigmented melanoma cells (SH-4) and two normal cell lines (BJ and HaCaT) showed a slight decrease of cytotoxicity for a conjugate, with low selectivity index (0.71 vs. 1.49 for GaPc). The present study suggests that the ability of collagen hydrolysate to form gels minimizes the high dark toxicity of GaPc. Collagen used for conjugation of a photosensitizer might be an essential step in advanced topical PDT.

## 1. Introduction

Photodynamic therapy (PDT) is a long-standing unconventional curative method, characterized by fast response after treatment [1,2]. PDT is based on the proper interaction between a photosensitizer (PS), light within red visible or near infrared spectra (630–850 nm), primary oxygen and other biomolecules in the media surrounding the PS [3,4]. The procedure starts with PS introduction to the treated cells, after proper time for PS uptake in the cells and irradiated with light, consistent with the PS absorption maxima, which leads to the PS conversion to triplet excited state. This further initiates the photochemical reactions with formation of reactive oxygen species but the highly cytotoxic singlet oxygen [5]. PDT has the benefit of a local treatment with low overall tissues’ toxicity and the general lack of side effects.

An intensive absorption in the far-red spectral region (>680 nm), which does not overlap the spectra of the endogenous natural cellular chromophores appears an appropriate characteristic of phthalocyanine dyes, which makes them favored potential PS drugs [6,7,8]. Phthalocyanine complexes (MPcs) in PDT method are Type II—photosensitizers with singlet oxygen generation. Presently, there are small number of the clinically approved phthalocyanine complexes for treatment of solid tumor localizations [9]. The further research and development of novel MPcs derivatives is still actual, considering a few drawbacks such as relatively low selectivity, high dark cytotoxicity and harsh phototoxicity provoking undesirable effects on the normal adjacent cells [10].

Collagen has the function as transporting molecules in the distribution of many tissues’ native physiological constituents such as growth factors, genes, proteins, etc. as well as an effective carrier in drug delivery formulations [11,12,13]. The several types of tissue collagen are known for their in vivo light absorption, biocompatibility with bioactive substances, low immunogenicity, and hemostatic characteristics. Collagen can form natural associates with significant impacts on their biological functionality. As a transporter demonstrates many advantages over other natural and synthetic compositions, such as promising degradability, high contact surface, minimal toxicity, and high compactness of cationic-charged amino groups [14].

The interaction of photosensitive drugs with native bioorganic molecules, part of circulating body fluids, is known as valuable approach for medication delivery with high impact on the therapeutic outcomes [15]. PDT process may influence collagen cross-linking and accelerate collagen biosynthesis, under the condition of sufficient UV-visible light [16]. There are additional advantages of utilization of collagen for PDT being its adsorptive ability for the hydrophobic compounds, the minimal risk of developing resistance and finally the expand uptake, stability, and retention in physiological conditions. There are many studies about the effects of native collagens of different origin for drug delivery, but a few in relation with photosensitizers and PDT [17]. Moreover, the impact of collagen hydrolysate over the phthalocyanine photosensitizer for PDT is still unknown. Furthermore, we could expect that the negative effects on healthy tissues caused by the photosensitization reactions will minimize by the presence of collagen. The identified photophysical parameters of collagen include a very intensive absorption band in the UVC region (190–260 nm) and a noticeable, characteristic fluorescence in the wide spectral range (300–700 nm) which depends on the substantial collagen modifications and the excitation wavelength [18,19]. These characteristics do not overlap the optical properties of the studied phthalocyanines. In addition, these exogenous photosensitizers cannot induce unfavorable photochemical processes of collagen photobleaching, for example in the skin exposed to therapeutic red light. Yova and coauthors showed that the photosensitization with natural dye hypericin tends to increase the photobleaching of collagen under UV-visible spectrum of irradiation [20]. In the same study the authors reported that an addition of hypericin resulted in shift of the collagen fluorescence to longer wavelengths. This early study also reported formation of the matrix protein cross-links due to PDT, which is hindering an invasive cellular migration. The denatured collagen (gelatin) demonstrates spectra characteristic for the breakdown of crosslinks in collagen after UV spectrum of exposure. Similar observations were for hypocretins after the addition to collagen, which resulted in quenching of collagen fluorescence and slight shift in its maximum. The changes in collagens’ fluorescence have application in fluorescence diagnosis of superficial tumors and other pathologies [21,22]. Most probably this phenomenon is due to fluorescence quenching of collagen by the mechanism of charge transfer between chromophores. This includes singlet oxygen interaction with amino acid residues, which tend the generation of other reactive species. The newly generated free radicals during PDT can react with surrounded biomolecules to form cross-links and inactivate the growth factors, which are increasing the resistance of enzymatic degradation of collagen [23].

The present study aims to synthesis, chemical and photophysical characterization, and evaluation of photodynamic efficacy of a new Ga (III)-phthalocyanine (GaPc). The bovine collagen hydrolysate (Clg), which tends to interact with GaPc was added to form conjugate with GaPc. The cytotoxicity of GaPc alone and as a conjugate with Clg were studied on pigmented melanoma cell line (SH-4) and two normal cell lines (human keratocytes cells, HaCaT and fibroblasts cells, BJ) in comparison. In addition, the photo-safety of both were evaluated on an embryonal mouse cell line (BALB/c 3T3, clone A31 cells).

## 2. Results and Discussion

**Scheme 1:**
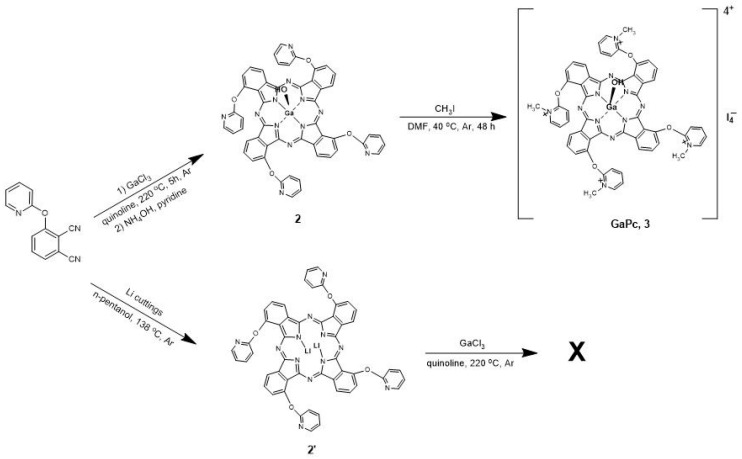
Synthetic pathways for synthesis of Gallium (III) phthalocyanine (GaPc, 3).

Gallium (III) phthalocyanine complex (GaPc) was successfully synthesized starting from a phthalonitrile (Scheme 1). Additional attempts were made with lithium or metal-free phthalocyanine to insert the atom of gallium but maybe because of the need of a high energy for the atom exchange, this pathway was not successful.

**Figure 1:**
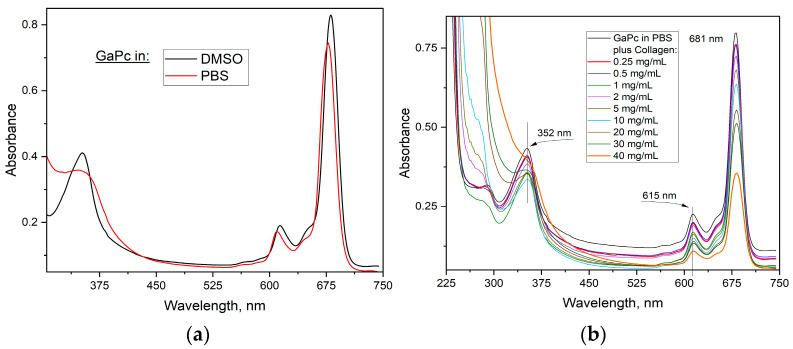
Absorption spectra of Ga(III)-phthalocyanine (GaPc) in: dimethylsulfoxide (DMSO) and phosphate-buffered saline (PBS) for 6 µM GaPc (**a**) and in PBS with hydrolyzed collagen (Clg) for a range of concentrations for a constant concentration of 6 µM GaPc (**b**).

The absorption spectra of GaPc were recorded in dimethylsulfoxide (DMSO) and a phosphate-buffered solution (PBS, pH 7.2), (Figure 1a). A characteristic Q band for non-aggregated phthalocyanines was observed in both media. The band in the UVA region was shown at 352 nm in water solutions and at 358 nm in DMSO. Two characteristic bands were observed in the visible region (615 nm and 686 nm, DMSO), with the low-intensity band at 610 nm or 615 nm and a strong sharp Q-band at 681 nm to 686 nm, depending on the solvents‘ polarity. All spectra strictly followed the Lambert–Beer law. The absorption maxima showed a small red shift of approx. 5–6 nm as compared to similar peripherally substituted phthalocyanines [24,25]. The absorption spectra showed that the addition of collagen hydrolysate (Clg) to GaPc (6 µM) diminished the Q-band and increased the Soret band for a range of Clg concentrations (0.25–40 mg/mL) (Figure 1b). The UV-band disappeared with the addition of Clg, with an increase in the Clg concentration. The peak was transformed to the shoulder, as was seen for the highest concentration (40 mg/mL) of the Clg linear macromolecules.

**Figure 2:**
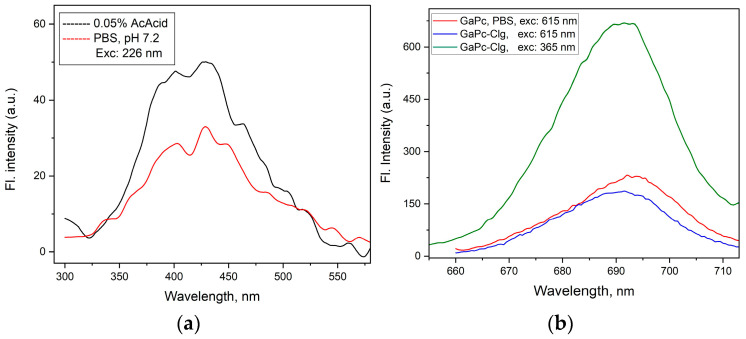
Fluorescence spectra: (**a**) collagen hydrolysate (Clg) in acetic acid (0.05% acetic acid) and phosphate-buffered saline (PBS, pH 7.2) at exc: 226 nm; and (**b**) conjugate with Clg (GaPc–Clg) at exc: 615 nm.

The fluorescence study was carried out upon excitation at 365 nm and 615 nm for the GaPc and its conjugate with the Clg. The emission peak of the GaPc was registered with a maximum at 704 nm (DMSO) and with a relatively high fluorescence quantum yield (0.23). The novelty was the fluorescence spectrum of the hydrolysate Clg which was evaluated with a wide non-uniform band at 400–450 nm for several excitations’ wavelength (exc: 226 nm, 260 nm and 266 nm), (Figure 2a). This band is typical for phenylalanine which may be the main fluorescent amino acid in the studied hydrolysate. The emission band of GaPc in the PBS was recorded at 691 nm, which by the addition of Clg the maximum moved slightly towards the blue region (Figure 2b). At an excitation wavelength of 365 nm, an increase in the emissions was shown for the GaPc in the conjugate. This was suggested to be due to the contribution of Clg in the fluorescence emission of GaPc. The fluorescence quantum yield of GaPc in conjugate GaPc–Clg was determined to have low value (0.012) at the typical excitation wavelength (615 nm) for phthalocyanine. The photophysical studies showed that GaPc was characterized by red-shifted absorption and fluorescence wavelengths (686 nm and 704 nm, DMSO) due to the non-peripheral substitutions. In addition, GaPc showed alower intensity and blue-shifted spectra in the water medium with the addition of Clg.

**Figure 3:**
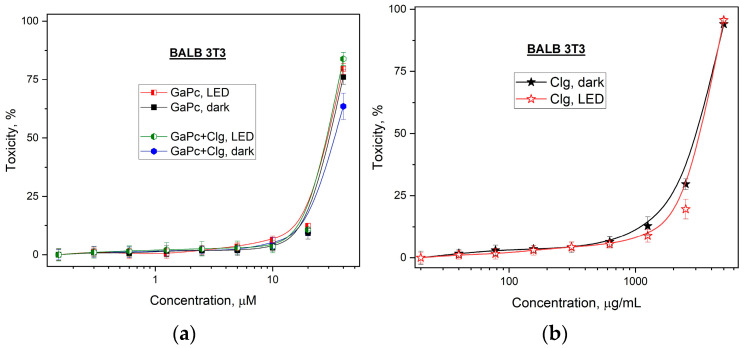
Dark and photo-cytotoxicity: (**a**) Ga (III) phthalocyanine (GaPc), bovine collagen hydrolysate (Clg) and the conjugate of both (GaPc–Clg), and (**b**) Clg, all studied on embryonal BALB 3T3 cell line (exposure with solar light-emitting diode, LED 360 nm–850 nm).

The normal cell line BALB 3T3 showed a similar behavior for concentrations up to 10 µM for the samples, with and without 660-nm light exposure (Figure 3). The lack of a cytotoxic effect on BALB 3T3 cells was observed for concentrations of GaPc and GaPc–Clg, which usually are usable in PDT. Collagen was evaluated for concentrations 100 times higher without the influence of the applied light (Figure 3b). The evaluation of photo- and dark cytotoxicity of Ga (III)-phthalocyanine (GaPc) and hydrolyzed collagen (Clg), and the freshly formed conjugate (GaPc–Clg) was carried out in dark conditions and with a specific light irradiation (LED 660-nm). The photo-safety study was carried out on a model mouse embryo fibroblasts normal cell line BALB 3T3 (Figure 3).

There was no difference in the obtained curves for GaPc in both treatment conditions (Figure 3a). These results suggested a good photo-safety with respect to solar light-emitting diode (LED) irradiation. A lack of dark and phototoxicity was shown for Clg for the study concentration range (Figure 3b). As can be seen, the cytotoxic effect was observed for concentrations much higher than 10 mg/mL, which are usually taken as supplements. The dark toxicity of GaPc was registered with a slight decrease after solar LED irradiation of the tested cells.

Table 1 summarizes the calculated CC_50_ values for the photo- and dark cytotoxicity for evaluation of the photo-safety properties and the photo-irritation factor (PIF) of three tested photosensitive substances on the embryonal BALB 3T3 cell line. The photosensitizer GaPc was evaluated with relatively high values of 33.90 μM and 29.51 μM, and with PIF = 1.15 which is typical for non-toxicity in solar light-exposure compounds. The data for the conjugate GaPc–Clg showed similar values (30.55 μM and 29.11 μM) and PIF = 1.05 which was also favorable, and suggested the high photo-safety impact of the collagen (0.95) as well as the conjugate (1.05). The results showed that the conjugation with a collagen hydrolysate with a spectrum in the UV region that did not lower the high photo-safety of the compound.

**Figure 4:**
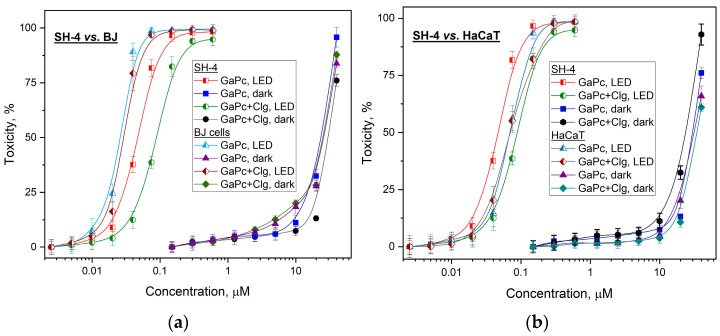
Dark and photo-cytotoxicity of Gallium(III) phthalocyanine (GaPc), and the conjugate GaPc–Clg on melanoma cell line (SH-4) versus normal cell lines BJ (**a**); and HaCaT (**b**). Light from a light-emitting diode (LED) at 660 nm with a light dose of 50 J/cm^2^.

The photodynamic activity studies were carried out to compare GaPc and its conjugate (GaPc–Clg) on human melanoma cancer cells (SH-4), (Figure 4). In addition, two normal cell lines with different origins, namely the skin keratocytes (HaCaT) and the fibroblasts (BJ), were investigated. The same treatment conditions were applied in order to evaluate the selectivity index (SI) of both structures. A relatively high phototoxicity of the GaPc was observed for low concentrations (0.01–0.1 µM) and for this concentration range, GaPc was evaluated without dark toxicity for the SH-4 tumor cell line as well as for a normal BJ cell line (Figure 4a). The phototoxicity results were obtained upon irradiation with PDT-specific parameters of a light-emitting diode (LED) with a maximum of 660 nm, a power density of 60 mW/cm^2^, and a light dose of 50 J/cm^2^, which were previously reported to have high potentiation [26]. GaPc was evaluated with a high phototoxic effect on tumors but also for the normal cell line HaCaT (Figure 4b). It was observed to have >50% cellular phototoxicity for very low concentrations (0.05–0.1 µM).

The main photo- biological parameters were calculated on the base of the obtained cytotoxicity results (Table 2). As seen, the characteristic values for a photosensitizer were evaluated for photother-apeutic index (PI = 819) for melanoma tumor cell line (SH-4). In case of normal cell lines (HaCaT and BJ) this index (PI) showed diverse values namely 580 and 1208. The results for a conjugate GaPc-Clg showed value of PI for tumor cells which are approx. two times lower (PI = 360) as compared to the values for GaPc alone (PI = 819). The normal cell line HaCaT and BJ cell line showed the same tendency for PI values of GaPc-Clg. This suggests the high photodynamic efficiency of GaPc as a photoactive agent. However, GaPc-Clg showed low PI = 360 which reduced the general toxicity. There was no sig-nificant difference in the PI values for both normal cell lines with less than 10% lower PI for GaPc-Clg as compared to GaPc. The calculated selectivity index (SI) has a value of 1.49 which is approx. double that for GaPc-Clg towards HaCaT cells (0.71). The similar tendency was observed for BJ cells (0.28). The high dark toxicity of GaPc was observed for concentrations almost ten times higher than a needed to obtain optimal photodynamic effect y towards SH-4 pigmented melanoma cells.

**Figure 5:**
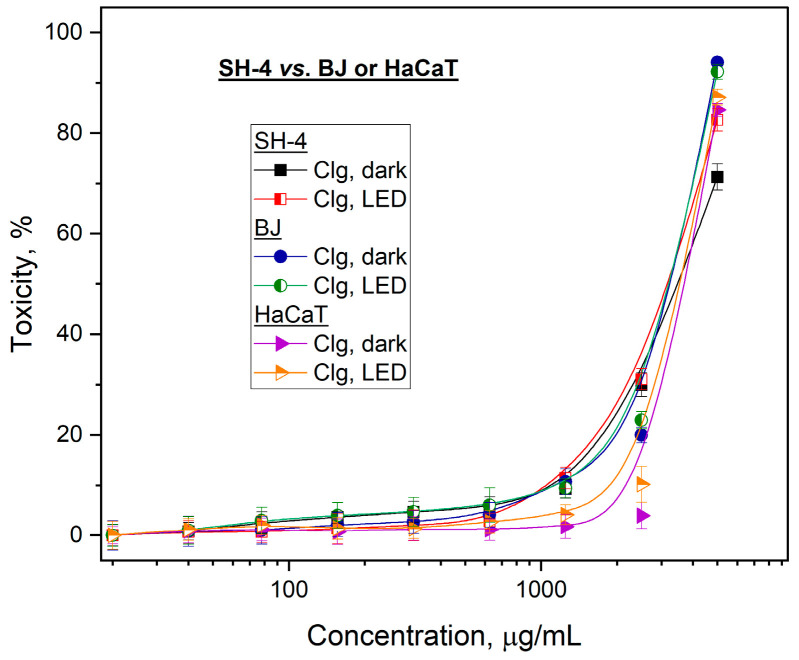
Dark and photo-cytotoxicity of bovine collagen hydrolysate (Clg) on SH-4 vs. BJ and HaCaT. Irradiation from a light-emitting diode (LED) at 660 nm with a light dose of 50 J/cm^2^.

The studies with Clg showed low cytotoxicity, which also diminished the effect of the conjugate in the case of normal keratinocytes HaCaT and fibroblasts cells BJ. Furthermore, the photo-cytotoxic effect of GaPc was determined to be relatively strong on the human melanoma tumor cell line (PI = 819), which was reduced by two times after the conjugation for GaPc–Clg (PI = 360). However, GaPc–Clg was evaluated with a low value of the selectivity index because of the Clg, which tended to lower cellular toxicity (Figure 5).

The present study focuses on the influence of a bovine collagen hydrolysate (Clg) used for supplement on the main properties of absorption and fluorescence, and the photobiological properties for evaluation of the photodynamic activity of a new complex such as Gallium (III) phthalocyanine (GaPc) towards human pigmented melanoma cells (SH-4). The photosensitizer (GaPc) was designed as cationic and hydrophilic, with non-peripheral substitution groups based on our previous efforts with other metallophthalocyanines (MPcs) [24,25,26]. The synthesis protocol followed the well-developed synthetic pathway [24,25]. Some attempts were made by using a substituted lithium phthalocyanine complex or metal-free phthalocyanine as starting compounds, but this pathway was not productive. A reason could be the big atom size of gallium which needs high energy for coordination in the closed ring macrocyclic molecule.

The studies on photophysical properties of GaPc in dimethylsulfoxide (DMSO) and water buffer solutions (PBS and 0.05% acetic acid) showed bathochromic shift of the absorption bands (686 nm to 681 nm in DMSO and PBS) as compared to the similar peripherally substituted GaPc1 [24]. These observations were expected because of the non-peripheral substitution groups of GaPc as was well documented for other phthalocyanines [26]. The formation of the conjugate with collagen (GaPc-Clg) was registered with decrease of absorption Q-band of GaPc and the increase with loss of shape of Soret band (Figure 1b). The fluorescence study was carried out in organic and water solutions (Figure 2). The emission spectra showed the fluorescence band with lower intensity maximum and a hypochromic shift to 694 nm because of the water media and the presence of collagen. In addition, a quenching of fluorescence of GaPc because of Clg was observed as compared to the emission of GaPc as pure com-pound (fluorescence quantum yield of 0.012 and 0.23).

The studied GaPc was evaluated with a high photodynamic activity as compared to similar phthalocyanine complexes studied on pigmented melanoma cells [27,28,29]. A high photo-safety (low cytotoxicity) was determined for GaPc and for its conjugate on the tested model embryonal cell line BALB 3T3 for the concentrations which related to PDT usage (Figure 3a). A low cytotoxic effect was also observed for collagen used as the food supplement at concentration range which is higher than used for human consumption (Figure 3b). The studies showed no dependence on the applied irradiation (LED 660 nm) as seen from the overlapping of the curves for both treatment conditions. This observation can be explained by the suitable spectrum of the light source with maximum at 660 nm. The effects of collagen hydrolysate on the selectivity index (SI) and the phototherapeutic index (PI) showed a photo-safety of GaPc-Clg without influence on the normal cell line BALB 3T3 (Table 1).

The photo- and dark toxicities of GaPc showed the high values for the tested melanoma tumor cell line (SH-4) but with a significant concentration gap (*p* < 0.001 and *p* < 0.01) between results for the normal cell lines (BJ and HaCaT), (Figure 4a,b). The formed conjugate GaPc-Clg was evaluated to de-crease both dark and photo- cytotoxicity as was determined for normal cell lines. The cytotoxic effect was diminished with collagen addition as a positive effect of the natural collagen hydrolysate. The effect of collagen as seen is to lower dark toxicity of GaPc, which is a desirable property for the com-pounds used as photosensitizers (Table 2). On the other hand, this tends to provoke a reduction of harsh photo-cytotoxicity action of GaPc for the studied concentration range on normal cells. The results obtained for normal cell lines followed a similar tendency. These observations arise from the quenching behavior of collagen hydrolysate which tend to form gel at concentration over 10 mg/mL. This leads to lower the photo- properties of GaPc because of the limitation of the light absorption due to large polymeric-like structure of collagen macromolecules. The harsh effect of another photosensitive compound (hypericin) was observed at 355 nm and 532 nm laser irradiation with the bleaching of collagen fluorescence because of the irreversible decomposition, which should be taken in consideration for UV-actions on human skin [20]. The topical application of the gel-formulated drugs brings an advantage of fast response with high photo-safety for the PDT procedure.

Malignant melanomas are highly metastatic and life-threatening tumors. Several limitations of the outcome of the well-defined therapeutic clinical route have been reported [30]. Melanomas as highly metastatic tumors have critical outcome by application of the well-defined clinical route of therapeutic procedures has been reported with several limitations [31]. A major challenge in treating pigmented melanoma is the presence of melanin which protects pigmented cells from oxidative stress, changes cell metabolism, induces immune suppression and mutagenesis of microenvironment, thus protecting malignant melanocytes from various treatments [32]. In order to avoid an adverse reduction in PDT efficacy due to the absorption of light by melanin, combination with depigmentation may be necessary. In vivo studies demonstrated that combining hypericin-mediated PDT with depigmentation agents, such as tyrosinase inhibitors (kojic acid) or phenyl thiourea, significantly increases ROS production and decreases viability of MEL-1 cells to a similar extent as that of A375 cells, suggesting that this treatment increases susceptibility of melanoma cells [33]. Further, melanoma cells were treated by photobleaching in combination with PDT and 420-nm violet light [34], and the results showed that the bleaching effect of violet light on melanoma cells significantly increased their sensitivity to PDT. Therefore, a drug with the ability to inhibit melanin production or induce depigmentation would be an important component in the therapeutic arsenal to treat melanoma more effectively. Previous studies with different photosensitizers including metallophthalocyanines (MPcs) suggested their potential on highly metastatic melanoma cells treated with PDT methodology [35]. The early-stage treatment of pigmented melanoma can be treated with PDT with a low frequency of resistance. The local character of the method allows to diminish the general toxicity and to improve the drug-penetration into the malignant surface. The present study suggested that these properties can diminish by the addition of biomolecules such as collagen hydrolysate with well-approved therapeutic impact on human health.

## 3. Conclusions

A novel non-peripheral tetra- methylpyridiloxy substituted Ga(III)-phthalocyanine (GaPc) and its freshly formed physical conjugate with bovine hydrolyzed collagen (GaPc-Clg) were studied for photodynamic therapy (PDT) of pigmented melanoma cells. GaPc was successfully synthesized using the pathway from a monomer. The studies on photophysical properties suggested that collagen lowers the values of the absorption and fluorescence of GaPc alone. For example, conjugation was observed with a blue shifted absorption (681 nm) and a lower intensity Q-band at the maximum of PDT irradiation, and the fluorescence with a lower quantum yield (0.012). The photo- and dark cytotoxicity study on tumor cells were observed with higher values for GaPc than for its Clg conjugate. For example, the photo-safety was examined as optimal (0.95, 1.05 and 1.15) on the tested BALB 3T3 cells. An approx. > 100 times concentration gap was determined between the dark and photo- cytotoxicity on both tested normal cell lines (keratocytes HaCaT and fibroblast BJ). The hydrolyzed collagen was unaffected by the light exposure (solar LED and LED 660 nm) for all tested cell lines. A diminish dark toxicity was observed for the conjugate GaPc-Clg with the selectivity index twice higher for GaPc alone than for the conjugate. Generally, it can be concluded that the used bovine collagen hydrolysate tends to reduce the photo- properties and the efficiency of the studied photosensitizer Ga(III)-phthalocyanine but it has the positive effect of reducing the harsh cytotoxicity on the normal cells which could be damaged during the light exposure.

## 4. Materials and Methods

### 4.1. Chemicals and Synthesis

Gallium (III) phthalocyanine with methylpyridiloxy groups (GaPc) was synthesized following the modification of a previously developed scheme [24,25]. All used solids were dried before usage. The solvents were additionally purified. The bovine collagen hydrolysate (Clg) had a certificate for human consumption as a food supplement. It has the characteristics of type III collagen (with traces of type I collagen) and was used as received from the vendor (Article Coll B^TM^ 3000 SE, Ter Apelkanaal, DCP B.V., Groningen, The Netherlands).

#### 4.1.1. 1(4),8(11),15(18),22(25)-Tetrakis-[(2-pyridiloxy) phthalocyaninato] hydroxygallium(III) (2)

The reaction mixture of 3-(2-pyridiloxy)phthalonitrile (1.0 g, 4.52 mmol) and anhydrous gallium(III) chloride (0.200 g, 2.26 mmol) was stirred in freshly distilled quinoline (3 mL) under argon and heated at 220 °C for 5 h. The mixture was cooled to room temperature. Then, a mixture of pyridine and ammonia hydroxide (1:1, *v*/*v*) was added. The reaction continued by stirring under argon at 25 °C. The product was determined by an increase in solubility. After cooling, the reaction mixture was poured into hexane to obtain a fine dark-greenish precipitate. The isolation of the solid was obtained by centrifugation with several steps. The initial purification was carried out with hexane until the indication of the transparency of the supernatant. The product was purified on SiO_2_ using the mixture DCM-MeOH (9:1). Yield: 0.110 g (53%). IR [ν_max_/cm^−1^]: 3091 (Ar-CH), 1648 (C=C), 1562, 1532, 1520, 1397, 1327, 1287, 1258, 1132, 1112 (C-O-C), 1045, 805, 744. ^1^H-NMR (300 MHz, CDCl_3_): δ, ppm 8.64–7.24 (14H, m, Pc-H and Pyridyl-H), 7.68–6.81 (14H, m, benzene H). MALDI-TOF-MS *m*/*z*: Calc. 971.61 for C_52_H_29_N_12_O_5_Ga; Found [M]^+^ 971.16; [M + Na]^+^ 993.12.

#### 4.1.2. 1(4),8(11),15(18),22(25)-Tetrakis-[(*N*-methyl-2-pyridiloxy) phthalocyaninato] hydroxygallium(III) Iodide (GaPc, 3)

Phthalocyanine, 2 (0.100 g, 0.1 mmol) was dissolved in dry dimethylformamide (5 mL) and an excess of methyliodide (2 mL) was added stepwise. The reaction mixture was stirred under argon at a temperature of 40 °C for 48 h. The product was isolated by precipitation in chloroform. The formed fine water-soluble solid was washed with chloroform, dichloromethane, ethyl acetate, acetone, n-hexane, and hot ethanol and was isolated by centrifugation. The green solid was dried under vacuum over phosphorous pentoxide at 80 °C overnight. Yield: 0.80 g (78%). UV-Vis (DMSO) λmax/nm (log ε): 358 (3.43), 615 (3.28), 686 (4.26). Yield: 0.080 g (67%). IR [ν_max_/cm^−1^]: 3076 (Ar-CH), 1635, 1572 (C=C), 1531, 1471, 1326, 1223 (S=O), 1178, 1105 (S=O), 1026 (C-O-C), 931, 835, 764, 661 (S-O). ^1^H-NMR (300 MHz, DMSO-*d_6_*): δ, ppm 7.99–7.24 (28H, m, Pc-H and Pyridyl-H), 4.39 (12H, m, CH_3_). MALDI-TOF-MS *m/z*: Calc. 1539.34 for C_56_H_41_N_12_O_5_I_4_Ga; Found 385.83 [(M + 4)/4]^+^.

### 4.2. Photo-Physicochemical Study

The absorption spectra were measured using a spectrophotometer (Evolution 300 UV-VIS) with quartz cuvettes with a 1.0 cm optical pathway. A stock solution of GaPc (~2 mM) in Dimethylsulfoxide (DMSO) was freshly prepared. Clg was dissolved to a concentration between 2–10 mg/mL in phosphate-buffered saline (PBS) with a pH of 7.2 just before the measurements. The absorption spectra of GaPc alone were recorded in a range of 250–750 nm. The spectra of Clg were recorded in the UV region (190–350 nm) in the PBS. The study of the interaction between GaPc and Clg was registered by a titration of 6 µM GaPc in PBS, pH 7.2 with an increase of Clg concentrations or the opposite titration. After a short incubation time, the full spectrum (190–750 nm) was recorded. Fluorescence measurements were carried out using a fluorimeter Perkin-Elmer LS 55 (Basel, Switzerland). The spectra of GaPc were recorded at excitation wavelengths of 615 nm or 365 nm and emissions between 660–750 nm for constant concentration in DMSO or PBS. The emission spectra of GaPc–Clg were recorded at different excitation wavelengths (226 nm; 260 nm; 365 nm and 615 nm).

### 4.3. Photobiology Study

The phototoxicity studies were performed on four model cell lines. The solution of GaPc in DMSO was prepared as a stock: 2 mM. The cells were incubated after serial dilution with the culture medium to final concentrations between 0.0025–40 µM GaPc. The solutions of Clg and the conjugate GaPc–Clg were prepared in PBS (10 mg/mL Clg).

#### 4.3.1. Cell Lines and Cultivation

Cell lines BALB/c 3T3 clone A31 (ATCC^®^ CCL-163^TM^)-mouse embryo fibroblasts, BJ (ATCC^®^ CRL-2522™)-normal skin fibroblasts and SH-4 (ATCC^®^ CRL-7724™)-human skin melanoma, were all obtained from American Type Cultures Collection (ATCC, Manassas, VA, USA). Keratinocyte cell line HaCaT (CLS, cat. No. 300493) was obtained from CLS Cell Lines Service GmbH (CLS, Eppelheim, Deutschland). The cells were cultured in 25 cm^2^ and 75 cm^2^ tissue-culture flasks in DMEM-high glucose (4.5 g/L), 10% FBS, 2 mM glutamine and antibiotics (penicillin 100 U/mL and streptomycin 100 µg/mL) at 37 °C, 5% CO_2_ and 90% relative humidity. Cells were plated in a 96-well microtiter plate at a density of 1 × 10^4^ cells/100 µL/well and were incubated for 24 h. Before treatment with the test compounds, dry compounds were dissolved in DMSO and further diluted in the culture medium, so that the final concentration of DMSO was less than 1% (*v*/*v*). The incubation was carried out for a concentration range (0.0025–40 µM) of the GaPc as well as for the conjugate.

#### 4.3.2. Light Source and Parameters

A light-emitting diode LED 660-nm used as the light source was a product of ELO Ltd., Sofia, Bulgaria. The light source was fixed for a power density of 100 mW/cm^2^ and a light dose of 50 J/cm^2^, which is optimal for PDT studies. The photo-safety study was carried out with a solar simulator of a light-emitting diode (LED) Helios-iO (SERIC Ltd., Tokyo, Japan). The fluence rate was fixed by measurements of the power (power-meter PM 100D with a sensor S120VC, Thorlabs Inc., North Newton, KS, USA). The working dimension in the range 50 nW–50 mW was for the spectrum 200–1100 nm. The spectrum was measured within the range of 360–850 nm [26]. The intensity was achieved for a distance of 25 cm with a normal diffusion in the experimental zone of ≈1.16 W/m^2^.

#### 4.3.3. Photo- and Cytotoxicity Study

The complex GaPc and its conjugate GaPc-Clg were tested based on Neutral Red Uptake in vitro test (NRU-assay). This color method for determination of the cells’ viability in vitro is based on the capability of the alive cells to accumulate the Neutral Red dye in their lysosomes. The mouse embryonal fibroblast cells (BALB/c 3T3, clone A31) were cultivated in dishes with an area of 75 cm^2^ as monolayer cells’ cultures at the standard conditions. The cells with density 1 × 10^4^ cells in 100 μL culture medium per well were cultivated using a standard 96 well plate. The standard conditions were applied for the cell incubation for 24 h to reach a good adhesion. The cells were incubated after application of double increase of the treatment concentrations for each compound. The study was performed in two plates at the same time to have the dark control in identical conditions as the light irradiated for comparison of photo and dark cytotoxicity. One plate with cells containing GaPc or GaPc-Clg was kept in a dark place (covered with Al-foil) and was used for evaluation of dark toxicity. The second plate was irradiated with LED 660 nm with light dose of 50 J/cm^2^ and was used for evaluation of photo- toxicity. 24 hours after irradiation, the culture medium was changed to a medium containing the NR dye. Three hours later the wells were washed with phosphate buffered saline (PBS, pH 7.4) and a mixture of distil water/ethanol/acetic acid (50:49:1) was added. The measurements of optical density were carried out on TECAN microplate reader at λ = 570 nm. The cellular toxicity was calculated by the Equation (1):

Cytotoxicity (%) = (1 − (OD_570_ (treated sample)/OD_570_ (negative control)) × 100 (1)

Other parameters calculated based on the photo- and dark cytotoxicity studies are following the Equations reported before [27,28,29,30,31,32,33,34]. Briefly, these are the phototherapeutic index (PI), cytotoxicity (CC50), photodynamic activity (PDA50), selectivity index (SI), and photo-irritation factor (PIF) calculated using the Formula (2):

PIF = CC50 (−Irr)/CC50 (+Irr)(2)

where −Irr is the absence of light and +Irr is the presence of light.

Phototherapeutic index (PI) is defined as the ratio of CC50 (−Irr) (half maximal cytotoxic concentration) to PDA50 (half maximal photodynamic activity concentration) values and is used for a determine of light-induced potency [36,37,38,39]. The ratio between the CC50 (−Irr) value of the resting and the PDA50 value of the activated compound must be as high as probable. The PI value was calculated using the Equation (3).

CC_50_ (−Irr)/PDA_50_ (3)

The anticancer activity of GaPc and its cytotoxicity towards normal cell lines were used to calculate the SI value. For evaluating any anti-cancer activity of a sample, its cytotoxicity against non-malignant cell line must be determined to calculate the SI value [40,41,42]. SI value > 10 was assumed to belong to a selected potential sample that can be further investigated. Weerapreeyakul et al. [43] proposed a lower SI value (>3) for classifying of a prospective anti-cancer sample. Rashidi et al. [44] considered that the SI values more than 2 were as high selectivity. The SI value was calculated by Equation (4).

SI = CC_50_ of non-malignant cell line/CC_50_ of tumor cells (4)

### 4.4. Statistics

The experiments were carried out in triplicate and the data are presented as a mean value ± standard deviation (SD) by the Student’s t test. The difference between the two means was compared by an unpaired Student’s t test. Values of *p* < 0.05 were considered to be significant.

## Figures and Tables

**Table 1:** Toxicity and photo-irritation factor (PIF) of Ga(III)-phthalocyanine (GaPc), bovine collagen hydrolysate (Clg) and the conjugate (GaPc–Clg) expressed by CC_50_ values ± SD (μM) for exposure of embryonal BALB 3T3 cell line with solar light-emitting diode (LED) with a dose of 10 J/cm^2^.

Compounds	Cytotoxicity	Phototoxicity	PIF *
Clg **	3148.63 ± 80.48	3299.40 ± 67.79	0.95
GaPc	33.90 ± 1.78	29.51 ± 0.44	1.15
GaPc–Clg	30.55 ± 0.63	29.11 ± 0.38	1.05

* PIF < 2 not phototoxic, 2 ≤ PIF < 5 probable phototoxicity, PIF ≥ 5 phototoxic, ** Clg [μg/mL].

**Table 2:** Photo- and cytotoxicity of Ga(III)-phthalocyanine (GaPc), bovine collagen hydrolysate (Clg) and the conjugate (GaPc–Clg) expressed by CC_50_ values of the mean ± SD (µM).

Cell lines	Compounds	Cytotoxicity	Phototoxicity	PI *	SI 1 **	SI 2 ***
tumor SH-4	Clg	3507.38 ± 111.38	3227.80 ± 63.26	1	1.11	1.02
	GaPc	30.06 ± 0.4150	0.0367 ± 0.0016	819	1.49	0.54
	GaPc–Clg	27.74 ± 0.8150	0.0770 ± 0.0035	360	0.71	0.28
HaCaT	Clg	3716.47 ± 36.82	3583.67 ± 69.74	1		
	GaPc	32.67 ± 1.37	0.0548 ± 0.0059	596		
	GaPc–Clg	31.61 ± 0.47	0.0545 ± 0.0045	580		

* Phototherapeutic index (PI), ** Selective index (SI1) calculated vs. HaCaT, *** Selective index (SI2) calculated vs. BJ cell line.

## Data Availability

The experimental data are available at request of the corresponding author.
